# Silent neonatal influenza A virus infection primes systemic antimicrobial immunity

**DOI:** 10.3389/fimmu.2023.1072142

**Published:** 2023-01-24

**Authors:** Anna Sophie Heinemann, Jan Lennart Stalp, João Pedro Pereira Bonifacio, Filo Silva, Maike Willers, Julia Heckmann, Beate Fehlhaber, Lena Völlger, Dina Raafat, Nicole Normann, Andreas Klos, Gesine Hansen, Mirco Schmolke, Dorothee Viemann

**Affiliations:** ^1^ Department of Pediatric Pneumology, Allergology and Neonatology, Hannover Medical School, Hannover, Germany; ^2^ Department of Microbiology and Molecular Medicine, University of Geneva, Geneva, Switzerland; ^3^ Institute of Immunology, University Medicine Greifswald, Greifswald, Germany; ^4^ Department of Microbiology and Immunology, Faculty of Pharmacy, Alexandria University, Alexandria, Egypt; ^5^ Institute for Medical Microbiology and Hospital Epidemiology, Hannover Medical School, Hannover, Germany; ^6^ Cluster of Excellence RESIST (EXC 2155), Hannover Medical School, Hannover, Germany; ^7^ Center for Inflammation Research, Faculty of Medicine, University of Geneva, Geneva, Switzerland; ^8^ Translational Pediatrics, Department of Pediatrics, University Hospital Würzburg, Würzburg, Germany; ^9^ Center for Infection Research, University Würzburg, Würzburg, Germany

**Keywords:** neonate, influenza A virus, influenza vaccination, innate immunity training, antimicrobial immunity, *Staphylococcus aureus*, sepsis

## Abstract

Infections with influenza A viruses (IAV) cause seasonal epidemics and global pandemics. The majority of these infections remain asymptomatic, especially among children below five years of age. Importantly, this is a time, when immunological imprinting takes place. Whether early-life infections with IAV affect the development of antimicrobial immunity is unknown. Using a preclinical mouse model, we demonstrate here that silent neonatal influenza infections have a remote beneficial impact on the later control of systemic juvenile-onset and adult-onset infections with an unrelated pathogen, *Staphylococcus aureus*, due to improved pathogen clearance and clinical resolution. Strategic vaccination with a live attenuated IAV vaccine elicited a similar protection phenotype. Mechanistically, the IAV priming effect primarily targets antimicrobial functions of the developing innate immune system including increased antimicrobial plasma activity and enhanced phagocyte functions and antigen-presenting properties at mucosal sites. Our results suggest a long-term benefit from an exposure to IAV during the neonatal phase, which might be exploited by strategic vaccination against influenza early in life to enforce the host’s resistance to later bacterial infections.

## Introduction

Up to 5 million cases of acute respiratory infections and approximately 500,000 human deaths worldwide are attributable to the influenza virus infections annually (1). However, most influenza virus infections in human populations remain asymptomatic (2). Especially among infants, recent data indicate that the incidence of asymptomatic and non-respiratory influenza illnesses is high (3).

During the neonatal window of life, responses to new antigens, particularly microbial challenges, can have lifelong imprinting effects on the immune system (4–6). Therefore, it is of high interest to better understand which early-life microbial challenges alter or promote immune adaptation to acquire homeostasis and protective immune memory. The high rate of inapparent influenza virus infections during infancy raises the question of long-term immunological consequences from a silent neonatal exposure to an influenza A virus (IAV). Two landmark cohort studies have shown associations between severe illnesses from respiratory syncytial virus or human rhinovirus infection in the first year of life and the later development of asthma (7, 8). In mouse models, this association only held true for infections during the neonatal period (9). In contrast, IAV infection of murine pups rather protected against later airway hyperreactivity and allergic asthma (10, 11).

It is well known that influenza virus infections increase the risk of bacterial coinfections and secondary lung infections, particularly with Staphylococci and Streptococci (12). Moreover, respiratory IAV infections have repeatedly been shown to have remote effects altering the gut microbiota in human and murine adults (13–15). In part the alteration of bacterial growth conditions after IAV infection depend on viral manipulation of the antimicrobial host response. Notably, these models rely on adult animals. Whether and how early-life infections with IAV impact the postnatal development of systemic antimicrobial immunity is largely unexplored.

In the present study we used different mouse models of silent neonatal IAV infection and influenza vaccination to assess their impact on a later systemic infection with an unrelated pathogen, i.e., *Staphylococcus aureus* given intravenously. In contrast to the above cited secondary infection models, we observed an improved clearance of *S. aureus* in adolescence and adulthood after neonatal exposure to IAV, primarily due to a priming effect of IAV on antimicrobial innate immune functions. Evidence is provided that this priming effect is only achieved if IAV is encountered during the neonatal period but not beyond.

## Material and methods

### Viruses

Human H1N1 virus (A/Netherlands/602/2009, IAV) was described previously (16). This virus was kindly provided by F. Krammer, Icahn School of Medicine at Mount Sinai, New York, NY. Virus stocks were grown at 37°C for 48 h on Madin Darby Canine Kidney cells (MDCK). Virus titers of virus stocks were determined by standard plaque assay and indicated in PFU. Virus stocks were stored at -80°C. The live attenuated influenza vaccine (LAIV) was a 6:2 reassortant of A/Ann Arbor/6/1960ca (cold-adapted) expressing hemagglutinin (HA) and neuraminidase (NA) of A/Puerto Rico/8/1934. This virus was generated by reverse genetics and grown in MDCKII cells at 33°C as described previously (17). The pDZ rescue system was kindly provided by P. Palese, Icahn School of Medicine at Mount Sinai, New York, NY.

### Bacteria

*Staphylococcus aureus* strain Newman, GenBank accession number AP009351.1, was used and inoculated 1:100 in Todd-Hewitt-Bouillon (Roth, Karlsruhe, Germany). Bacteria were incubated at 37°C until the late-logarithmic phase was reached (OD_600 =_ 0.7-0.8). Suspensions were adjusted to a count of 1 × 10^10^ colony-forming units (CFU)/ml and stored at -80°C. The infection dose was controlled by serial dilutions and plating on blood agar (Oxoid, Wesel, Germany) for colony-counting.

### Mice and mouse models

C57BL/6J mice purchased from Charles River Laboratories (Sulzfeld, Germany) were housed under specific-pathogen-free conditions at the Central Animal Facility at the Hannover Medical School. Litters were used randomly at d3 or d10 of life for i.n. treatment with 5 PFU of IAV or 60 PFU of LAIV. Control mice received PBS i.n. Mice were evaluated twice daily according to an established score (18). In five IAV-treated mice, presence of IAV vRNA in lungs was analyzed by qRT-PCR on d10. Otherwise, mice were used 18 days (adolescence) or 53 days (adulthood) after IAV infection as indicated for sepsis induction by i.v. injection of 7.5 × 10^6^ CFU of *S. aureus* per gram of body weight. In all models, mice were weaned at d21 of life. Disease severity was assessed at 0, 12, 24, 36, 48, 64, 72 and 80 h by using a clinical score for neonatal mice (18) that was adapted for adolescent and adult mice. Score parameter used were fur appearance, posture, movement, agility, attention, anal area hygiene, dehydration and body weight changes in percent of initial weight. Every category was scored between 0 - 3 points with 3 points in a single category or 12 points total as dropout criteria. Cytokine and immunoglobulin studies in plasma samples and bacterial burden analyses in organs were performed 24 h and 80 h p.i., respectively. Bacterial burden in organs was determined as described previously by plating serial dilutions of homogenates of prior perfused organs (19). For flow cytometry studies, single-cell suspensions of lungs were prepared and lamina propria mononuclear cells (LPMCs) isolated from colons as previously described (20).

### Ethics statement

Mouse experiments were performed according to the German Animal Welfare Legislation and as approved by the Lower Saxony State Office for Consumer and Food Safety, Germany (approval no. 33.12-42502-04-17/2608).

### Quantitative real-time PCR

Total RNA was isolated from murine lung lysates using the NucleoSpin RNA II Kit (Macherey-Nagel, Dueren, Germany). cDNA was synthesized from 40 ng of total RNA using the SuperScript VILO Master Mix (Invitrogen, Carlsbad, USA). Viral RNA was reverse-transcribed and amplified using primers aligning to the RNA of the viral matrix protein M1 (forward 5’-AGATGAGTCTTCTAACCGAGGTCG-3’; reverse 5’-TGCAAAAACATCTTCAAGTCTCTG-3’). qPCRs were performed with iTaq SYBR Green (Bio-Rad, Hercules, CA; USA) and the M1 primer to determine viral M1 RNA (vM1). To quantify vM1 copies, a standard curve was performed by qPCR, using serial 10-fold dilutions of pDZ M1 plasmid. M1 copy numbers per lung lysate were calculated based on the linear regression of the standard curve.

### Analysis of cytokine, immunoglobulin and complement levels

Cytokine levels and concentrations of IgG1, IgG2a, IgG2b, and IgG3 in mouse EDTA plasma were measured by using murine LEGENDplex™assays (BioLegend, San Diego, CA, USA) according to the manufacturer´s instructions. FACS Canto II flow cytometer was used for measurements and the LEGENDplex™ Data Analysis Software v7.0 (BioLegend) for data analysis.

Levels of *S. aureus*-specific IgM were determined by ELISA as described previously (21). In brief, ELISA plates were coated overnight at 4°C with 50 μl of extracellular proteins of *S. aureus* NewmanΔ*spa* (cultivated in TSB; 10 μg protein/ml 5%SDS). After blocking, mouse plasma was added in a 1:3 serial dilution (dilution range: 1:50 - 1:12,150) in blocking buffer (Blocking Reagent for ELISA; Roche, 11112589001). Goat anti-mouse-IgM POD (Jackson ImmunoResearch, West Grove, PA, USA; 0.8 mg/ml) was used as a detection antibody at a dilution of 1:2,500. Optical density was measured at 450 nm using a Tecan infinite M200PRO (software: i-control 1.10). All measurements were performed in duplicates and the means were used for analysis. Antibody binding was calculated using the non-linear standard curve protocol for GraphPad Prism (EC50 × dilution factor_EC50_).

Levels of C3 and C3a in mouse EDTA plasma samples were determined using in-house ELISA described previously (22, 23).

### Flow cytometry

All antibody staining panels (**Supplementary Table 1**) included labelling with Fixable Viability Dye eFluor 506 or Fixable Viability Dye eFluor 780 (Thermo Fisher Scientific, Waltham, MA, USA) to exclude dead cells as well as anti-mouse CD16/CD32 (2.4G2; BioLegend) for blocking purposes. Staining was performed for 30 min at 4°C in the dark. After staining, cells were fixed with 2% PFA. For the analysis of lung immune cells, lung single-cell cell suspensions were stained with a leukocyte panel to determine CD11b^+^Ly6C^low^Ly6G^+^ polymorphonuclear neutrophils (PMN), CD11b^+^Ly6C^hi^Ly6G^-^ monocytes, CD19^+^ B cells, CD3^+^CD4^+^CD8^-^ T helper cells and CD3^+^CD4^-^CD8^+^ cytotoxic T cells, and with a myeloid cell panel to assess CD11b^+^F4/80^+^CD11c^-^SiglecF^+^ eosinophils, tissue-resident CD11b^low^F4/80^hi^CD11c^+^SiglecF^+^MHCII^+^ AMs, CD11b^+^F4/80^+^CD11c^+^SiglecF^-^Ly6C^+^MHCII^+^CD64^+^ IMs, and CD11b^+^F4/80^+^CD11c^+^SiglecF^-^Ly6C^-^MHCII^hi^CD64^-^ DCs. For the analysis of tissue-resident cells in the colon, isolated LPMCs were stained with a LPMP panel to detect F4/80^hi^CD11b^+/−^Ly6G^−^CD11c^−^ yolk sac–derived LPMPs and F4/80^+^CD11b^+^Ly6G^−^CD11c^−^ blood-derived LPMPs, and a Treg panel for the analysis of colonic FoxP3^+^CD3^+^CD4^+^CD25^+^ regulatory T cells. In the latter, after extracellular staining of CD3, CD4, and CD25, cells were fixed with 2% PFA and stained intracellularly with rat anti-mouse FoxP3 mAb (FJK-16s, eBioscience) in FACS buffer with 0.5% saponin and 0.2% Tween20 for 30 min at 4°C in the dark.

*Data acquisition and analysis*. All flow cytometry analyses were performed using a FACS Canto II flow cytometer (BD Biosciences). Data were analyzed using DIVA software v8.0.1 (BD Biosciences) and Kaluza software v2.1 (Beckman Coulter, Miami Lakes, USA). Gating of outlined cell types in cell suspensions stained with the antibody panels listed in **Supplementary Table 1** was done for intestinal cells as previously described (20) and for lung cells as illustrated in **Figure S1**.

### Analysis of phagocyte functions

For the assessment of *S. aureus* phagocytosis and killing after neonatal IAV challenge, murine blood-derived macrophages were enriched by seeding freshly prepared cell suspensions from spleens of neonatally IAV or PBS pretreated d21 mice, respectively, in tissue culture plates at a concentration of 2 × 10^6^ cells/ml in RPMI 1640 supplemented with glutamine, 25 mM HEPES, 10% fetal bovine serum but without antibiotic supplements. After overnight culture, non-adherent cells were removed, and adherent macrophages were infected with *S. aureus* Newman at a MOI of 10. To harvest bone marrow-derived macrophages (BMDMs) femurs were dissected from neonatally IAV or PBS pretreated d21 mice and flushed with cold RPMI 1640 supplemented with glutamine, 25 mM HEPES, 10% fetal bovine serum, and 1% antibiotic-antimycotic and stored in liquid nitrogen. Vials of freezed bone marrow were defrosted and resuspended in supplemented RPMI 1640 additionally supplemented 10% L-cell media (LCM) as a source of MCSF growth factor. Cells were seeded at 1 × 10^6^ cells/ml and differentiated for 3 days to yield mature macrophages. Then cells were switched to antibiotic-antimycotic-free medium for 24h and infected with *S. aureus* Newman at a MOI of 10. One hour p.i., cells were harvested to assess the number of phagocytosed intracellular bacteria. To determine the macrophages’ killing function, cultures were treated at 1 h p.i. with gentamicin at a final concentration of 100 μg/ml to kill possibly remaining extracellular bacteria. Subsequently, these cells were lysed 3 h (blood-derived macrophages only) and 6 h p.i. by adding sterile water. To determine the number of intracellular *S. aureus*, serial dilutions of cell lysates were plated on blood agar plates, and *S. aureus* colonies were counted after 18 h of incubation at 37°C.

### Analysis of plasma opsonizing activity

The opsonizing activity of plasma of neonatally IAV- or PBS-pretreated d21 mice was tested by preincubating *S. aureus* suspensions for 1 h with the respective plasma. Subsequently, these opsonized bacteria were used to infect macrophages from neonatally unchallenged juvenile (d21) C57BL/6J mice in the phagocytosis and killing assay.

### Bacterial lysis assay

Plasma-mediated bacterial lysis was tested by incubating 1 × 10^7^ CFU of heat-inactivated *S. aureus* for 1 min with heat-inactivated and non-heat-inactivated plasma from d21 mice that have been neonatally challenged with PBS or IAV. Afterwards, bacterial suspensions were washed and fixed for 30 min with 2% PFA. The number of bacteria was assessed flow cytometrically in the forward/side scatter. First, a gate was set capturing the bacterial population after complete flow-cytometric measurement of 1 × 10^7^ CFU of not plasma incubated heat-inactivated *S. aureus*. This gate was kept while measuring the corresponding samples of 1 × 10^7^ CFU of plasma-treated bacteria. The loss of bacteria was defined as the percentage of the number of gated untreated bacteria minus the number of bacteria left in this gate after plasma treatment from the number of untreated bacteria.

### Bacterial growth inhibition assay

In order to test plasma-mediated antimicrobial activity against living bacteria, suspensions of 7.5 × 10^7^ CFU *S. aureus* were incubated with plasma from d21 respective d28 mice that have been neonatally respective at d10 challenged with PBS or IAV at a volume ratio of 5: 1 in RPMI 1640 without supplements in 0.5 ml reaction tubes on ice. Control bacterial suspension were not treated with plasma. After 1h of incubation, serial dilutions of bacterial suspensions were plated on blood agar plates and colonies counted after 18 h of incubation at 37°C.

### Statistical analysis

Kaplan-Meier survival curves were generated using the Mantel-Cox test. To test for differences between the clinical course of *S. aureus*-mediated sepsis over time in the offspring of the control, IAV and LAIV groups, *post hoc* 2-way ANOVA Bonferroni tests were employed. For the comparison viral RNA loads in the lungs and cytokine levels after sepsis induction in the control and IAV groups at different time points, *post hoc* Kruskal-Wallis Dunn’s multiple comparison tests were used, respectively. Two-group comparisons for bacterial burden, leukocyte recruitment, immunoglobulin levels, complement levels, bacterial lysis, and the numbers and MHC-II expression of tissue-resident leukocytes in lungs and colons were performed by applying Mann-Whitney U (MWU) tests. To test for differences between the control and IAV group in the development of body weights, the bacterial phagocytosis and killing capacity of macrophages and the opsonizing plasma activity, ANOVA analyses across settings and *post hoc* Tukey’s multiple comparison tests between sub-settings were applied, respectively. p-values of < 0.05 were judged to be significant (*p < 0.05, **p < 0.005, ***p < 0.001).

## Results

### Silent neonatal exposure to IAV primes immunity against *S. aureus*

To investigate the impact of a silent neonatal exposure to IAV on the development of systemic antimicrobial immunity, we established a mouse model of subclinical IAV infection. After intranasal (i.n.) application of IAV at 5 plaque forming units (PFU) to murine neonates at day (d) 3 of life IAV was transiently detectable in the lungs on d10 but not anymore on d21 (**Figures S2A, B**). This challenge with IAV remained clinically silent, neither affecting the development of body weight (**Figure S2C**) compared to PBS-treated control mice.

**Figure 1:**
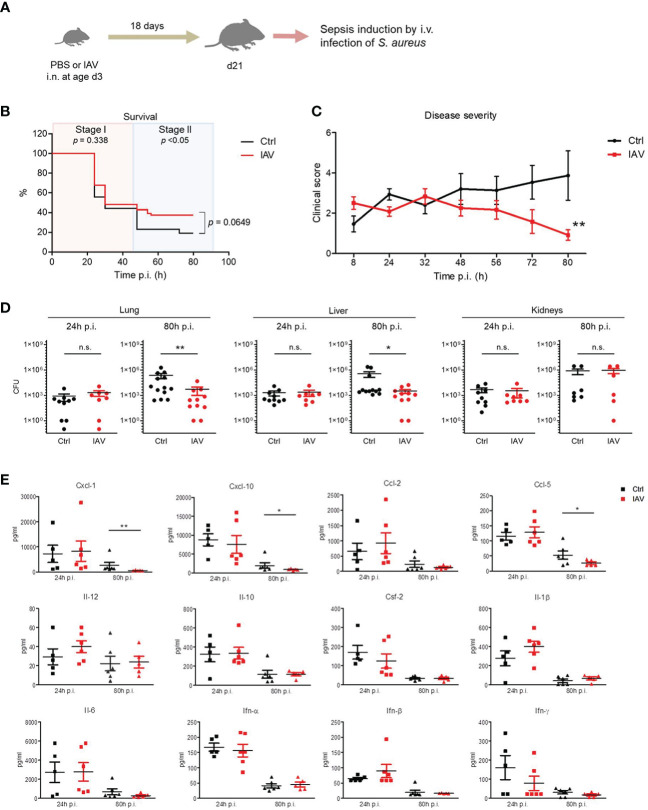
Control of *S. aureus* infections in juvenile mice is improved after silent neonatal exposure to IAV. Newborn mice were treated intranasally (i.n.) at d3 of life with PBS (control, Ctrl) or 5 PFU of the H1N1 virus (IAV). After 18 days, sepsis was induced in these mice by intravenous (i.v.) application of *S. aureus*. **(A)** Experimental setup. **(B)** Survival was observed for 80 h post-infection (p.i.) with *S. aureus* and plotted as percentages over time (Mantel-Cox test). **(C)** Clinical scores at indicated time points p.i., plotted as means ± SEM. **p < 0.01 (*post hoc* 2-way ANOVA Bonferroni test) (Ctrl n = 52 from 7 litters, IAV *n* = 56 from 7 litters). **(D)** Bacterial burden was determined in independent experiments in indicated organs at 24 h and 80 h p.i. (each group n = 6-13 from 2-7 litters, respectively). Plotted are means ± SEM, *p < 0.05, **p < 0.005 (MWU test). **(E)** Plasma cytokine levels at 24 h and 80 h p.i. (each group n = 5-6, each from 2 independent experiments). Plotted are means ± SEM, *p < 0.05, **p < 0.005 (*post hoc* Kruskal-Wallis Dunn’s multiple comparison test). n.s., not significant.

At adolescence (d21), sepsis was induced in neonatally IAV- and PBS-pretreated mice by intravenous (i.v.) injection of *S. aureus* (**Figure 1A**). In the overall sepsis survival, no significant differences could be observed (**Figure 1B**). However, from 48 h p.i. on the severity of the clinical courses of sepsis differed considerably and was significantly milder after neonatal exposure to IAV compared to IAV-naïve control mice (**Figure 1C**). When dissecting the survival data in more detail, we found that the first 48 h of sepsis were critical, deciding about survival/death in the majority of mice, irrespective of the neonatal treatment. Beyond that critical first sepsis phase, the death rate dropped down about one-half in the control group but even to one-fifth in the IAV group resulting in significant differential survival in this second stage of sepsis (**Figure 1A**, **Supplementary Table 2**). The improved survival and disease severity were accompanied by better pathogen control, with a 2 log 10 lower bacterial burden in lungs and livers at 80 h post infection (p.i.), while comparable at 24 h p.i. (**Figure 1D**). Similarly, the plasma cytokine response was comparable at 24 h p.i. but largely resolved at 80 h p.i. in IAV-pretreated mice. In contrast, systemic inflammation was still ongoing at 80 h p.i. in control mice as reflected by higher plasma levels of Cxcl-1, Cxcl-10 and Ccl-5 (**Figure 1E**).

To test how long the neonatal priming effect of IAV persists, we induced sepsis also in 8 weeks old (adult) mice following neonatal IAV exposure (**Figure S3A**). In contrast to juvenile mice, *S. aureus* induced a considerably milder sepsis disease in adult mice with little and no differential affection of the survival in both pretreatment groups (**Figure S3B**). While there was neither a clear difference in clinical disease scores (**Figure S3C**), the bacterial burden in lungs and livers were still significantly decreased in the IAV-primed group compared to the IAV-naïve group (**Figure S3D**).

Collectively, the data suggest that a neonatal exposure to IAV has remote effects promoting systemic antimicrobial functions, which are particularly crucial for the success of pathogen clearance and the clinical condition in the second stage of bacterial sepsis. The sepsis protection offered by neonatal IAV exposure with respect to bacterial clearance persists beyond childhood until adulthood, though the beneficial effects on survival and clinical course diminish with time due to an overall change of the course of sepsis in adulthood.

### Training of antimicrobial immunity by IAV is restricted to the neonatal period

**Figure 2:**
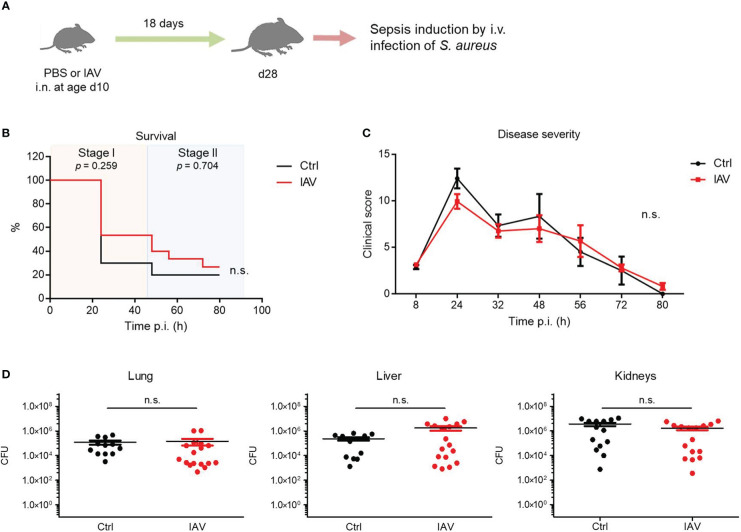
Exposure to IAV beyond the neonatal period has no impact on the later defense of *S. aureus*. Mice were treated i.n. at d10 of life with PBS (control, Ctrl) or 5 PFU of IAV. After 18 days, sepsis was induced in these mice by i.v. application of *S. aureus*. **(A)** Experimental setup. **(B)** Survival was observed for 80 h p.i. and plotted as percentages over time (Mantel-Cox test). **(C)** Clinical scores assigned at indicated time points p.i., plotted as means ± SEM (*post hoc* 2-way ANOVA Bonferroni test) (Ctrl n *=* 10, IAV n *=* 15, each from 2 litters). **(D)** Bacterial burden was determined in independent experiments in indicated organs 80 h p.i. (each group n *=* 12-17 from 7 litters, respectively). Plotted are means ± SEM. n.s., not significant (MWU test).

The neonatal window of life is particularly sensitive to impacts of environmental challenges on the developing immune system (4–6). Therefore, we investigated whether a subclinical IAV exposure after the murine neonatal period at d10 still has a beneficial effect on the later sepsis vulnerability during adolescence (**Figure 2A**). In this model, no significant differences were observed between the IAV group and the control group with regard to the sepsis survival (**Figure 2B**) or the clinical course of disease severity (**Figure 2C**). Consistently, the bacterial burden in organs at 80 h p.i. was comparable in *S. aureus* infected juvenile mice pretreated with IAV or PBS only on d10 (**Figure 2D**). Of note, the difference in the clinical scores of d28 and d21 mice are most likely attributable to an ongoing immune maturation beyond d21 (e.g., by expanding microbiota and/or introduction of solid food after weaning) with stronger cytokine responses (and consequently higher clinical scores in the first sepsis phase) but also more effective pathogen clearance in the second phase of sepsis. The latter is reflected by lower clinical scores and comparable bacterial burden at 80 h p.i. in the control groups in d28 compared to d21 mice despite significant larger organ sizes and higher body weights (average 7.3 g at d21 and 12.3 g at d28). Collectively, the data suggest that an exposure to IAV after the neonatal period at d10 remains without effect on systemic antimicrobial immunity.

### Persistent enhancement of phagocyte functions and recruitment after neonatal IAV priming

The observation of a long-term improvement of *S. aureus* control after silent neonatal exposure to IAV prompted us to examine more closely the impact of IAV on different cellular functions involved in the defense of *S. aureus*.

**Figure 3:**
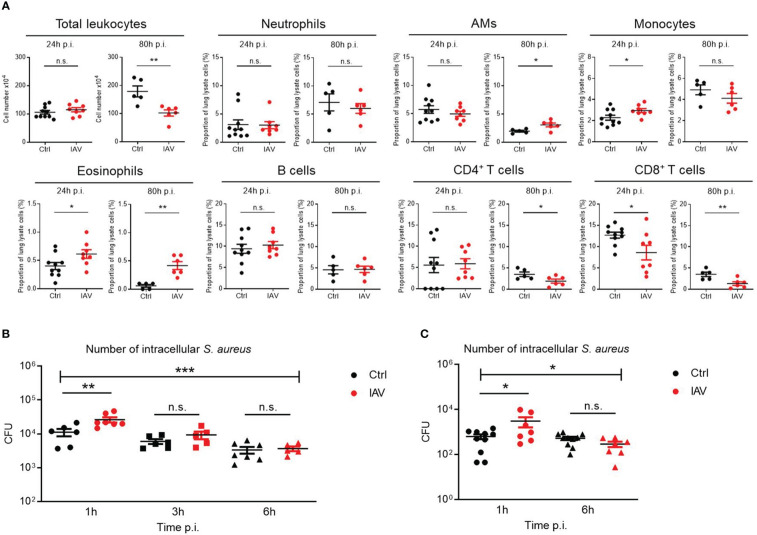
Silent neonatal IAV infections train phagocyte functions against *S. aureus* infections. Experimental setup as illustrated in **Figure 1A**. **(A)** After 24 h and 80 h of infection of d21 mice with *S. aureus*, the total number of leukocytes and the proportion of indicated leukocytes harvested from the lungs were determined by flow cytometry in the Ctrl and IAV pretreatment group (each group n *=* 5-10 from 2 litters, respectively). Plotted are means ± SEM, *p *<* 0.05 (MWU test). **(B, C)** Bacterial phagocytosis and killing capacity of blood-derived macrophages **(B)** and BMDMs **(C)** obtained from d21 mice after neonatal i.n. pretreatment with PBS or 5 PFU of IAV (each group n *=* 7-10 from 2 litters, respectively). Plotted are the numbers of colony-forming units (CFU) of intracellular *S. aureus* at 1 h, 3 h and 6 h (in blood-derived macrophages) respective 1 h and 6 h (in BMDMs) after *ex vivo* infection of macrophages at MOI 10. Bars represent means ± SEM. Significant differences were determined by ANOVA across settings (***p < 0.0001, *p < 0.05) and by *post hoc* ANOVA Tukey’s multiple comparison tests between pretreatment groups at resp. time points p.i. (**p < 0.005, *p < 0.05). n.s., not significant.

As the lung was the organ showing the largest reduction in bacterial load following neonatal IAV priming (**Figure 1D**), we compared the recruitment of leukocytes to the lungs of *S. aureus*-infected d21 mice. The number of total leukocytes 24 h after sepsis induction was comparable (**Figure 3A**). However, the proportions of monocytes and eosinophils were significantly higher in the lungs of neonatally IAV-pretreated mice compared to control mice. In contrast, alveolar macrophages (AMs), neutrophils, B cells, and CD4^+^ T helper cells showed no differences, while the proportion of CD8^+^ cytotoxic T cells was even lower in the IAV group (**Figure 3A**). After 80h of sepsis induction, the number of total leukocytes further increased in the PBS group but not in the IAV group. Thereby the proportions of AMs and eosinophils were significantly lower and the proportions of CD4^+^ T cells and CD8^+^ T cells higher in PBS-pretreated mice compared to IAV-pretreated mice (**Figure 3A**). This data suggests less involvement of adaptive immunity but an accumulation of phagocytes in IAV exposed mice. The discontinuation of leukocyte recruitment in the second stage of sepsis in IAV exposed mice is in line with the improved bacterial clearing (**Figure 1D**), while both bacterial defense and leukocyte recruitment are obviously ongoing processes in PBS exposed control mice at this stage.

To assess phagocyte and killing functions, murine blood-derived macrophages and BMDMs of neonatally IAV- or PBS-pretreated d21 mice were *ex vivo* infected with *S. aureus*. After one hour, the bacterial load of macrophages of IAV-pretreated mice was significantly higher than that of control mice, pointing to a higher phagocytosis rate (**Figures 3B, C**). Remaining extracellular bacteria were then cleared by gentamycin treatment to determine the killing rate of phagocytosed bacteria. Despite higher numbers of phagocytosed bacteria at 1 h p.i., macrophages of the IAV group ended up with comparable low numbers of vital intracellular bacteria at 6 h p.i. as control macrophages, suggesting improved net killing after neonatal IAV priming.

Together, these data demonstrate that a silent exposure to IAV during the neonatal period establishes improved recruitment of phagocytes to bacterial infection sites and enhanced phagocytosis and net killing of macrophages later in life.

### Antimicrobial plasma activity is persistently increased following silent neonatal exposure to IAV

**Figure 4:**
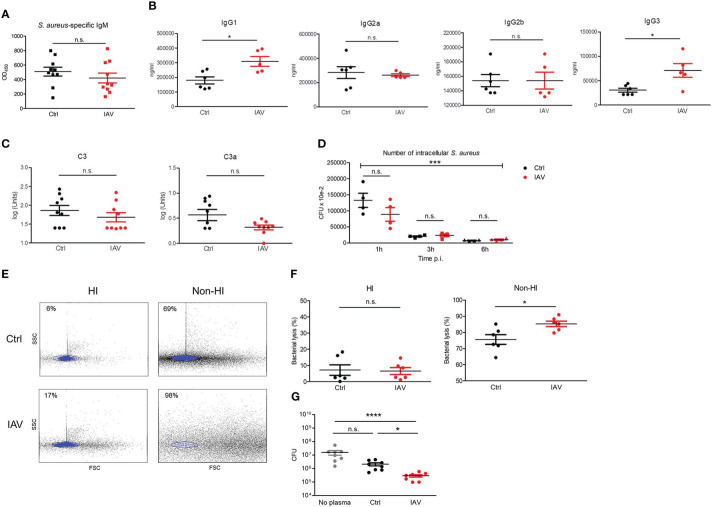
Silent neonatal exposure to IAV enhances humoral antimicrobial functions. Plasma was collected from d21 mice pretreated i.n. on d3 with PBS (Ctrl) or 5 PFU of IAV. **(A)** Levels of *S. aureus*-specific IgM as determined by ELISA. OD_450_, optical density measured at 450 nm (Ctrl n *=* 10, IAV n *=* 10). **(B)** Plasma concentration of indicated murine IgG subclasses (Ctrl n *=* 6, IAV n *=* 5). **(C)** Complement levels of C3 and C3a (Ctrl n *=* 9, IAV n *=* 9). Bars represent means ± SEM. *p < 0.05 (MWU tests). **(D)** Opsonizing plasma activity was tested by preincubating *S. aureus* with heat-inactivated (HI) plasma from d21 mice of the Ctrl or IAV group (each n *=* 4). Opsonized bacteria were used at MOI 10 for infection of macrophages obtained from untreated adult C57BL/6 mice. Plotted is the number of CFU of intracellular *S. aureus* at 1 h, 3 h and 6 h p.i. Indicated is the overall significance of *S. aureus* elimination over time (***p < 0.0001, ANOVA) and differences between the pretreatment groups at respective time points p.i. (*post hoc* ANOVA Tukey’s multaiple comparison tests). **(E, F)** Plasma-mediated bacterial lysis was determined flow cytometrically by analyzing the number of bacteria left after incubation of 1 × 10^7^ CFU of heat-inactivated *S. aureus* with HI plasma and non-HI plasma obtained from d21 mice of the Ctrl and IAV group. **(E)** Representative FACS scatter plots with the gate of intact bacteria highlighted in blue. Indicated are the percentages of bacterial lysis. **(F)** Bacterial lysis was plotted as percentage of bacterial loss after treatment with HI and not-HI plasma compared to not plasma treated bacteria (Ctrl n *=* 6, IAV n *=* 6). Bars represent means ± SEM, *p < 0.05 (MWU test). **(G)** CFU of *S. aureus* after 1h of growth without plasma and in the presence of plasma from d21 mice of the Ctrl and IAV group (each n *=* 8). Bars represent means ± SEM, *p < 0.05, ****p < 0.0001 (Kruskal-Wallis test with *post hoc* Dunn’s multiple comparison tests). n.s., not significant.

Immunoglobulins and complement are critical opsonizing factors that contribute to the elimination of bacteria. We next examined the impact of a silent neonatal IAV infection on humoral immune functions. Differences in *S. aureus*-specific IgM levels could be excluded in our model (**Figure 4A**). The measurement of total plasma levels of IgG subclasses showed significantly higher levels of IgG1 and IgG3 in the IAV exposed group compared to IAV-naïve mice (**Figure 4B**), while plasma levels for IgG2a and IgG2b were comparable (**Figure 4B**). Likewise, the levels of the complement factors C3 and C3a were comparable (**Figure 4C**). Functionally, no difference was detected for the total opsonizing activity of the plasma, since macrophages were able to phagocytose and kill *S. aureus* preincubated with heat-inactivated plasma from IAV-pretreated mice to the same extent as after preincubation with heat-inactivated plasma of PBS-pretreated control mice (**Figure 4D**). Total bacterial lysis activity of plasma was equally abrogated by heat-inactivation in both experimental groups. However, non-heat-inactivated plasma from neonatally IAV exposed d21 mice lysed heat-inactivated *S. aureus* bacteria (**Figures 4E, F**) and inhibited the growth of *S. aureus* bacteria (**Figure 4G**) to a significantly greater extent than non-heat-inactivated plasma from mice without neonatal exposure to IAV, suggesting increased plasma levels of antimicrobial peptides after neonatal IAV priming. Of note, non-heat-inactivated plasma from d10 IAV exposed d28 mice lysed heat-inactivated *S. aureus* bacteria and inhibited the growth of *S. aureus* bacteria to a comparable extent as non-heat-inactivated plasma from control mice (**Figure S4**), supporting that an IAV exposure beyond the neonatal period fails to prime the antimicrobial plasma activity.

These findings provide clear evidence that a silent neonatal exposure to IAV enhances the antimicrobial plasma activity but not the opsonizing plasma activity in the long term.

### Neonatal IAV priming promotes antigen-presenting properties of tissue-resident leukocytes

**Figure 5:**
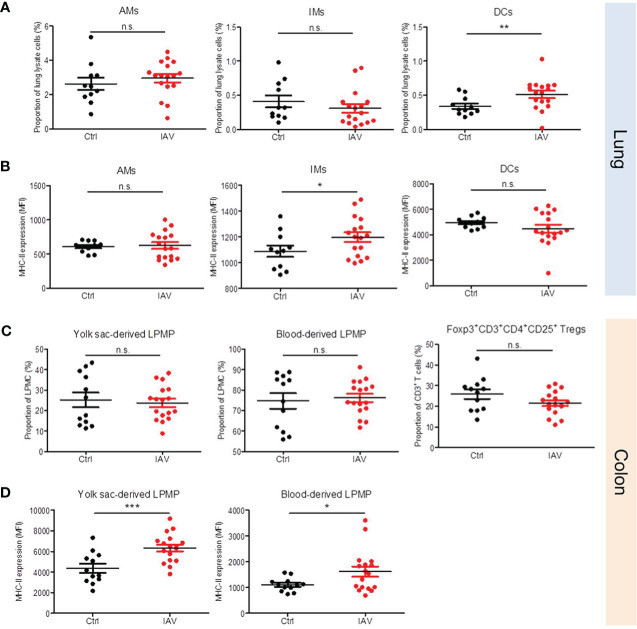
Neonatal exposure to IAV induces long-lasting changes in the phenotype of lung and gut resident leukocytes. Single-cell suspensions of **(A, B)** the lungs and **(C, D)** the colonic lamina propria from d21 mice pretreated i.n. with PBS (Ctrl, n *=* 11-12) or 5 PFU of IAV (n *=* 17) on d3 of life. **(A, C)** Numbers and **(B, D)** MHC-II expression (MFI, mean fluorescence intensity) of indicated leukocytes in lungs and colons. Bars represent means ± SEM. *p < 0.05, **p < 0.01, ***p < 0.005 (MWU test). n.s., not significant. AM, alveolar macrophage; DC, dendritic cell; IM, interstitial monocyte; LPMP, lamina propria macrophage; Tregs, regulatory T cells.

The broad priming effect of a neonatal IAV exposure on systemic antimicrobial immunity implicate possible changes also in the development of tissue-resident immune cells. We therefore profiled leukocytes isolated from the lungs and colons of neonatally IAV- or PBS-pretreated mice on d21 of life. No differences were found in the proportion of AMs and interstitial macrophages (IMs) in the lungs. In contrast, the proportion of dendritic cells (DCs) was significantly higher after neonatal exposure to IAV (**Figure 5A**). Moreover, IMs in the IAV group’s lungs showed a higher expression of MHC-II than in the control group (**Figure 5B**). Similar in the colon, there were no differences in the proportions of both yolk-sack derived and blood-derived lamina propria macrophages (LPMPs) and regulatory T cells (Tregs) (**Figure 5C**), however, MHC-II expression levels on yolk-sack derived and blood-derived LPMPs were significantly higher in IAV-pretreated mice than control mice (**Figure 5D**). Together, these findings suggest that a silent neonatal IAV infection influences also the development of tissue-resident innate immune cells, and promotes their antigen-presenting properties.

### Antimicrobial immunity can be primed by neonatal vaccination against influenza

**Figure 6:**
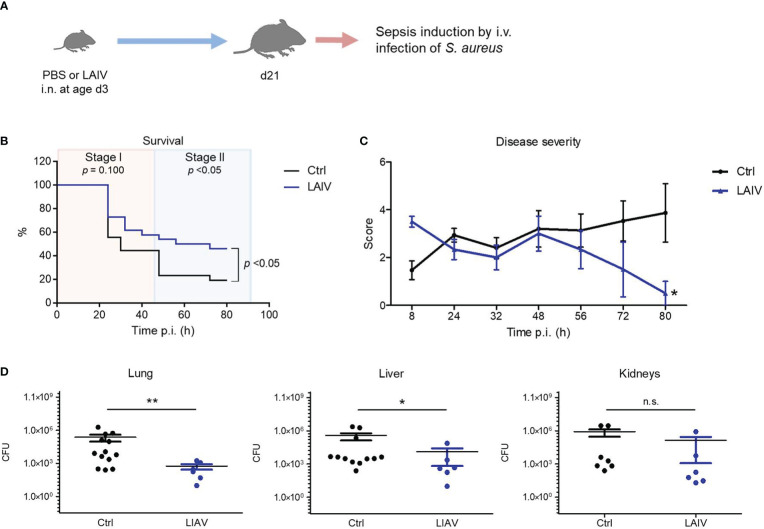
Neonatal influenza vaccination improves control of *S. aureus* infections beyond childhood. Newborn mice were treated i.n. at d3 of life with PBS (Ctrl) or a live-attenuated influenza vaccine (LAIV). After 18 days, sepsis was induced in these mice by i.v. application of *S. aureus*. **(A)** Experimental setup. **(B)** Survival was observed for 80 h p.i. and plotted as percentages over time. n.s., not significant (Mantel-Cox test). **(C)** Clinical scores assigned at indicated time points p.i., plotted as means ± SEM. *p < 0.05 (*post hoc* 2-way ANOVA Bonferroni test) (Ctrl n = 52 from 7 litters, LAIV n = 26 from 4 litters). **(D)** Bacterial burden was determined in independent experiments in indicated organs 80 h p.i. (each group n *=* 6-13 from 3 litters, respectively). Plotted are means ± SEM, *p < 0.05, **p < 0.005 (MWU test).

To take advantage of the discovered IAV priming mechanism as a preventive intervention strategy against bacterial infections, we next tested whether vaccination with a live attenuated influenza virus (LAIV) might prime the neonatal immune system in a similar fashion as an infection with a wild-type IAV. To this end, we inoculated neonates i.n. with a reassortant LAIV at 60 PFU (**Figure 6A**, **Supplementary Table 3**). Similar as the exposure to the wild-type IAV, neonatal vaccination with the LAIV improved the overall survival and in particular the survival during the second stage of a later induced *S. aureus* sepsis (**Figure 6B**). Accordingly, neonatal treatment with the LAIV also ameliorated the clinical course of a systemic *S. aureus* infection (**Figure 6C**) and improved the control of bacterial burden in organs (**Figure 6D**) compared to non-vaccinated control mice.

These data suggest that influenza vaccination with LAIV during the neonatal period has a persistent beneficial off-target effect on the postnatal maturation of systemic antimicrobial immunity, which might be exploited to enforce the h.ost’s resistance to later *S. aureus* infections.

## Discussion

**Figure 7:**
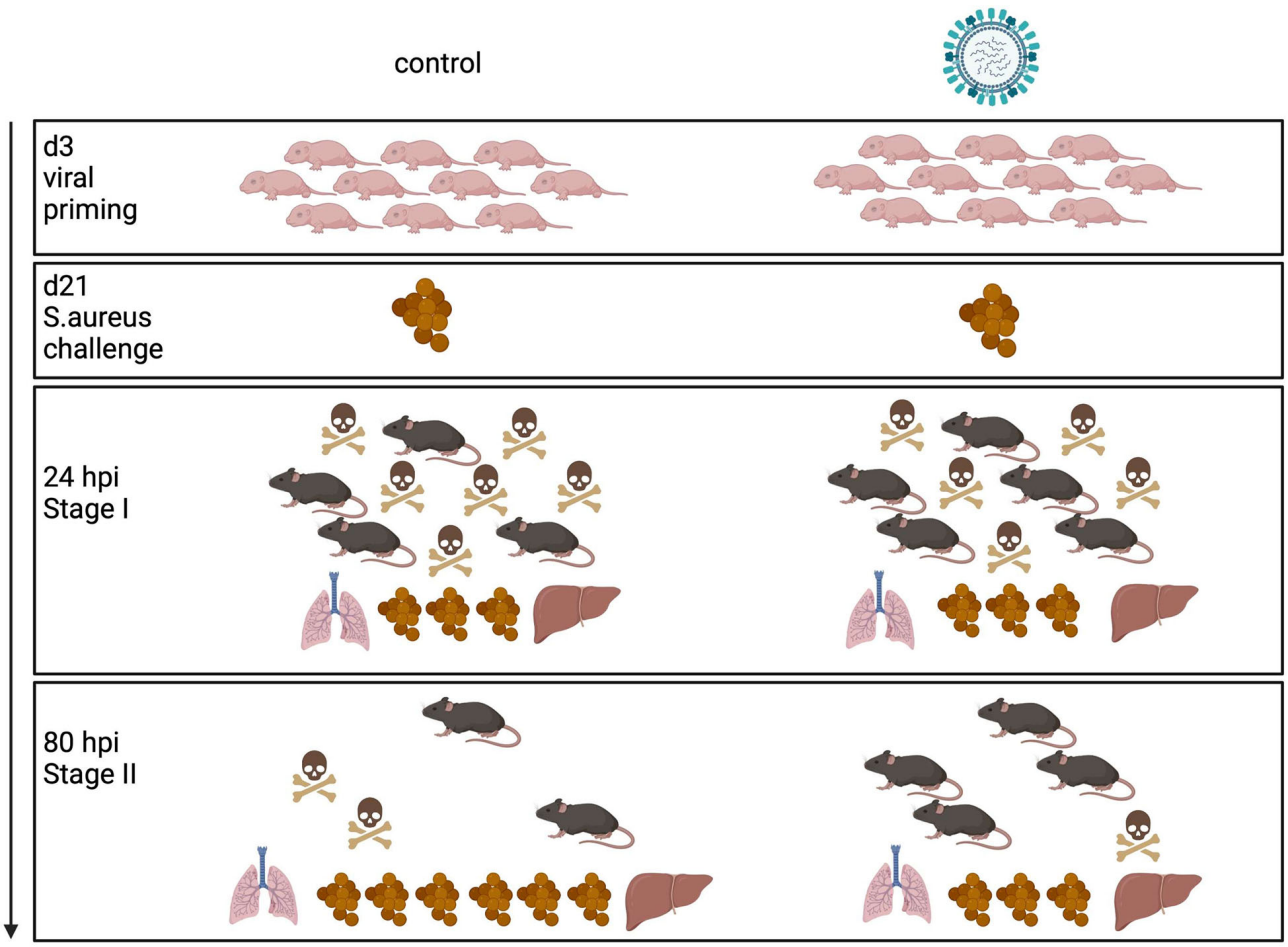
Model of innate antimicrobial priming by IAV exposure early in life. Graphical summary how a respiratory exposure of murine d3 neonates to IAV shapes their antimicrobial immunity in the long-term. While an early-life encounter of IAV does not influence the performance of the antimicrobial response during the acute early stage of a systemic *S. aureus* infection in adolescence (stage I), it improves pathogen clearance and survival during the second stage of sepsis (stage II). Control mice are depicted on the left side, mice challenged with IAV on d3 on the right side. The numbers of mouse icons are an approximation based on our experimental findings. Created with BioRender.

Influenza virus infections in infants can cause a broad clinical spectrum, ranging from severe to mild respiratory diseases to a probably underestimated number of non-respiratory and silent illnesses (3, 24). However, our knowledge of the long-term effects of neonatal influenza infections is rather limited. By using different mouse models, we have demonstrated for the first time that a respiratory exposure to IAV as well as a LAIV during the neonatal period have remote priming effects on systemic antimicrobial immunity against an unrelated bacterial pathogen. Summarized in a model (**Figure 7**) our specific findings were that a neonatal IAV exposure persistently enhances innate antimicrobial immune functions, which leads to improved pathogen clearance and, associated therewith, improved survival and disease courses during systemic *S. aureus* infections later in life. Follow-up studies will need to clarify whether early respiratory priming with IAV would also protect from sepsis with other gram-positive and gram-negative bacteria. Hitherto only fungal β-glucans have been shown to enhance non-specific resistance to *S. aureus* sepsis (25). Interestingly, this beneficial long-term effect of IAV is in stark contrast to its immediate local adverse impact that increases the risk of influenza virus-infected patients to life-threatening secondary respiratory bacterial infections.

The beneficial priming of antimicrobial immunity by the influenza virus resembles the heterologous effects of vaccines, particularly live attenuated vaccines such as Bacillus Calmette-Guérin (BCG), measles vaccine, and oral polio vaccine (OPV) (26–28). Such non-specific vaccine effects are believed to cause the increase of overall childhood survival in vaccinated individuals (29–32), suggesting protection against unrelated microbial challenges. Certain non-specific effects have also been found in LAIV-vaccinated human adults, including an altered *in vitro* cytokine response to unrelated pathogens (33), however the biological relevance was unclear so far. A recent study now revealed that the quadrivalent inactivated influenza vaccine has a heterologous effect on systemic anti-viral immunity by demonstrating a vaccine-induced transcriptional reprogramming of blood monocytes, which constrained the systemic inflammatory responses to SARS-CoV-2 (34). Here, we provide clear first *in vivo* evidence for heterologous effects of the influenza virus and influenza vaccine on systemic antibacterial immunity.

The beneficial effect of neonatal H1N1 influenza infection seems not to be strain-specific as the same effect could be observed for the vaccine strain, which has a different backbone (H2N2). However, it would be of interest to elucidate how specific the respiratory priming of systemic immunity, particularly against *S. aureus*, is to influenza viruses. Though the aggravating effect of early-life RSV infections on the risk later asthma (35, 36) contrasts the asthma protective effect of previous IAV infections (10, 11), it appears unlikely that the education of systemic *S. aureus* immunity *via* the respiratory route is specific to IAV given the known broad imprinting impact of respiratory microbial exposures on local antimicrobial immunity (e.g., by respiratory viruses other than IAV (37) or the respiratory microbiota including specific pathogen-associated molecular pattern molecules thereof (38, 39) and inhaled probiotics (40).

In our murine study, a sustainable reinforcement of antimicrobial immunity against *S. aureus* was achieved only if the IAV exposure took place during the neonatal period. This finding corroborates the considerable plasticity of the neonatal immune system in response to microbial interactions shown by others (5, 6). The timing of IAV infection also matters in the context of its effect on allergic sensitization, as IAV infection of 2-week-old suckling pups, but not adult mice, can protect adults from OVA-induced airway inflammation (10). Similarly, only a neonatal challenge with poly I:C, but not a challenge at day 8 anymore, induced long-lasting changes in the frequency and transcriptomes of non-lymphoid tissue resident Tregs (41). Though under certain conditions, antimicrobial immunity can also be long-term enhanced in adult mice, e.g., local immunity, specifically lung macrophages, by respiratory adenovirus infections (37) and IAV infections (42) or the stem cell niche by systemic administration of BCG (43). Differences in the maturity of mucosal and systemic immunity, innate as well as adaptive, and/or the dynamic changes in respiratory microbiota states are likely the reason for differential dealing of IAV in d3 and d10 mice that explains the failure of IAV to impact on *S. aureus* immunity after the neonatal period. Profound changes in the respiratory microbiota during the first days of life have a strong impact on immune maturation processes during this period, particularly on anti-viral immunity (44–46). Furthermore, multiple studies provided evidence for specific immune maturation processes during the first 2 weeks of a mouse life, such as increased epithelial production of antimicrobial peptides (47), DC functions (48, 49), frequency of lung resident T cells (50) and AMs (51), and advanced Th1 differentiation (48, 52). Considering that *S. aureus* is the leading cause of infection in the setting of critical illness and injury, our finding of enhanced anti-*S. aureus* immunity after neonatal IAV exposure might add an important argument to the research area of neonatal vaccinology. Currently, influenza vaccination is not recommended for infants younger than six months despite the absence of serious adverse events (53).

Multiple reports suggest that immune imprinting effects during the neonatal period are dependent on the gut microbiota (54–56). In adult animals, influenza virus infection induces profound alterations of the gut microbiota (13–15, 57). No change was observed in the adult murine gut microbiota composition after LAIV vaccination (14). We recently found long-lasting changes in the gut microbiota composition, associated with increase in fat mass, following infection of neonatal mice with a sub-lethal dose of a low pathogenic H5N1 IAV strain (58). Whether LAIV vaccination in neonates alters the developing gut microbiota and whether IAV- and LAIV-induced microbiota changes in turn contribute to the priming effect of IAV and LAIV on systemic antimicrobial immunity needs to be elucidated on the future.

Regarding the mechanism of the persistent effect of a neonatal IAV exposure on antimicrobial immunity, our study suggests induction of immunological memory at multiple levels of the innate immune system (59). Neonatal IAV exposure induced long-term macrophage phenotypes with increased antigen-presenting properties of tissue-resident macrophages in the lung and gut and an enhanced phagocytosis and killing capacity of blood-derived macrophages and BMDMs towards *S. aureus* bacteria. Innate *in vivo* training of bacterial killing capacity is a phenomenon otherwise described in murine herpesvirus latency (60). Moreover, we found an enhanced, heat-labile antimicrobial plasma activity after neonatal IAV priming, suggesting a proteinous nature of the active compounds. Several cationic peptides displaying varying degrees of activity against *S. aureus* are expressed by human leukocytes and epithelial cells (61, 62), and also in abundance by the human microbiome (63, 64). Furthermore, microbiota metabolites are essential regulators of the expression level of host defense peptides (65). The change in antimicrobial peptide expression levels after neonatal IAV infection might be attributed to direct and indirect cellular reprogramming as well as a shaping of the microbiota composition, which both might also explain the improvement of antigen-presenting properties that we observed at barrier sites. Prolonged presentation of influenza antigens after viral clearance is a well-described phenomenon (66), suggesting that IAV can induce a persistent general enhancement of antigen-presenting functions. Neonatal exposure to IAV might also lead to a microbiota development that reinforces the microbiome’s major regulatory role on the number and function of antigen-presenting cells (67).

In summary, our preclinical models demonstrate for the first time that a silent respiratory infection of neonates with the influenza virus induces remote priming effects on the developing innate immune system, which collectively allow for a better defense of systemic *S. aureus* infections beyond childhood. The latter could also be induced by treating neonates with a LAIV, wherefore early vaccination of infants against the influenza virus - apart from its target effect - might provide a strategic long-term benefit by enforcing the host’s resistance to later *S. aureus* infections. We would like to stress that the priming model we used here is very mild and consists only of a single asymptomatic viral infection. In contrast, human neonates undergo quite a series of respiratory infections within the critical imprinting window. Future studies will have to address the long-term effect of combinatorial infections. The fast-developing mouse model, might however not be ideal for this research question. A careful translation of our findings to human neonate imprinting will be essential.

## Data availability statement

The original contributions presented in the study are included in the article/**Supplementary Material**. Further inquiries can be directed to the corresponding author.

## Ethics statement

The animal study was reviewed and approved by Lower Saxony State Office for Consumer and Food Safety.

## Author contributions

AH, JS, MS and DV contributed to conception, design and methodology of the study. AH, JS, BF, JB, FS, MW, JH, LV, DR, NN and AK performed experiments. AH, JS and DV wrote the first draft of the manuscript. AH, JS, MW, DR, AK, GH, MS and DV contributed to manuscript revision and editing. GH, MS and DV acquired funding. All authors read and approved the submitted version.
