# Supplementary material for: Silent neonatal influenza A virus infection primes systemic antimicrobial immunity
Source: Front Immunol. 2023 Jan 24;14:1072142. doi: 10.3389/fimmu.2023.1072142 (PMC9902881; doi:10.3389/fimmu.2023.1072142)
Supplement: Supplementary file 1 [file Table_1.pdf]

Supplementary Table 1. Flow cytometry antibody staining panels

Target	Conjugate	Source	Reactivity	Clone	Company
Leukocyte panel lung cells					
CD11b	APC	rat	mouse	M1/70	BioLegend
CD3e	PE-Cy7	hamster	mouse	145-2C11	BD Biosciences
CD19	PE	rat	mouse	1D3	BD Biosciences
CD4	FITC	rat	mouse	6k 1.5	BD Biosciences
CD8a	PerCP-Cy5.5	rat	mouse	53-6.7	BD Biosciences
Ly6C	Pacific Blue	rat	mouse	HK1.4	BioLegend
Ly6G	BV510	rat	mouse	1A8	BD Biosciences
Myeloid cell panel lung cells					
CD11b	APC	rat	mouse	M1/70	BioLegend
CD11c	PerCP-Cy5.5	hamster	mouse	HL3	BD Biosciences
F4/80	PE	rat	mouse	BM8	eBioscience
Siglec F	PE-Cy7	rat	mouse	ES22110D8	Miltenyi Biotec
Ly6C	FITC	rat	mouse	HK1.4	BioLegend
MHC II	BV510	rat	mouse	M5/114.15.2	BioLegend
CD64	BV421	mouse	mouse	X54-5/7.1	BioLegend
Lamina propria macrophage (LPMP) panel intestinal cells					
CD45	FITC	rat	mouse	30-F11	eBioscience
F4/80	APC	rat	mouse	BM8	eBioscience
CD11b	APC-Cy7	rat	mouse	M1/70	eBioscience
CD11c	PerCP-Cy5.5	hamster	mouse	N418	Thermo Fisher Scientific
Ly6G	PE-Cy7	rat	mouse	1A8	BioLegend
Treg panel intestinal cells					
CD45	eFluor450	rat	mouse	30-F11	eBioscience
CD3e	PerCP-Cy5.5	hamster	mouse	145-2C11	eBioscience
CD4	eFluor780	rat	mouse	RM 4-5	eBioscience
CD25	PE Cy7	rat	mouse	PC61	BD Biosciences
Foxp3	APC	rat	mouse	FJK-16s	eBioscience
