# Supplementary material for: Silent neonatal influenza A virus infection primes systemic antimicrobial immunity
Source: Front Immunol. 2023 Jan 24;14:1072142. doi: 10.3389/fimmu.2023.1072142 (PMC9902881; doi:10.3389/fimmu.2023.1072142)
Supplement: Supplementary file 3 [file Table_3.pdf]

Supplementary Table 3. Survival and death rates during *S. aureus* -mediated sepsis stage I (≤48 h p.i.) and stage II (>48 h p.i.) following neonatal LAIV vaccination

	Ctrl	LAIV
Total number	52	26
Survival	10 [19%]	12 [46%]
Death <48 h p.i.	29 [56%]	11 [42%]
Death ≥48h p.i.	13 [25%]	3 [12%]
