# Supplementary figures and images for: Silent neonatal influenza A virus infection primes systemic antimicrobial immunity
Source: Front Immunol. 2023 Jan 24;14:1072142. doi: 10.3389/fimmu.2023.1072142 (PMC9902881; doi:10.3389/fimmu.2023.1072142)

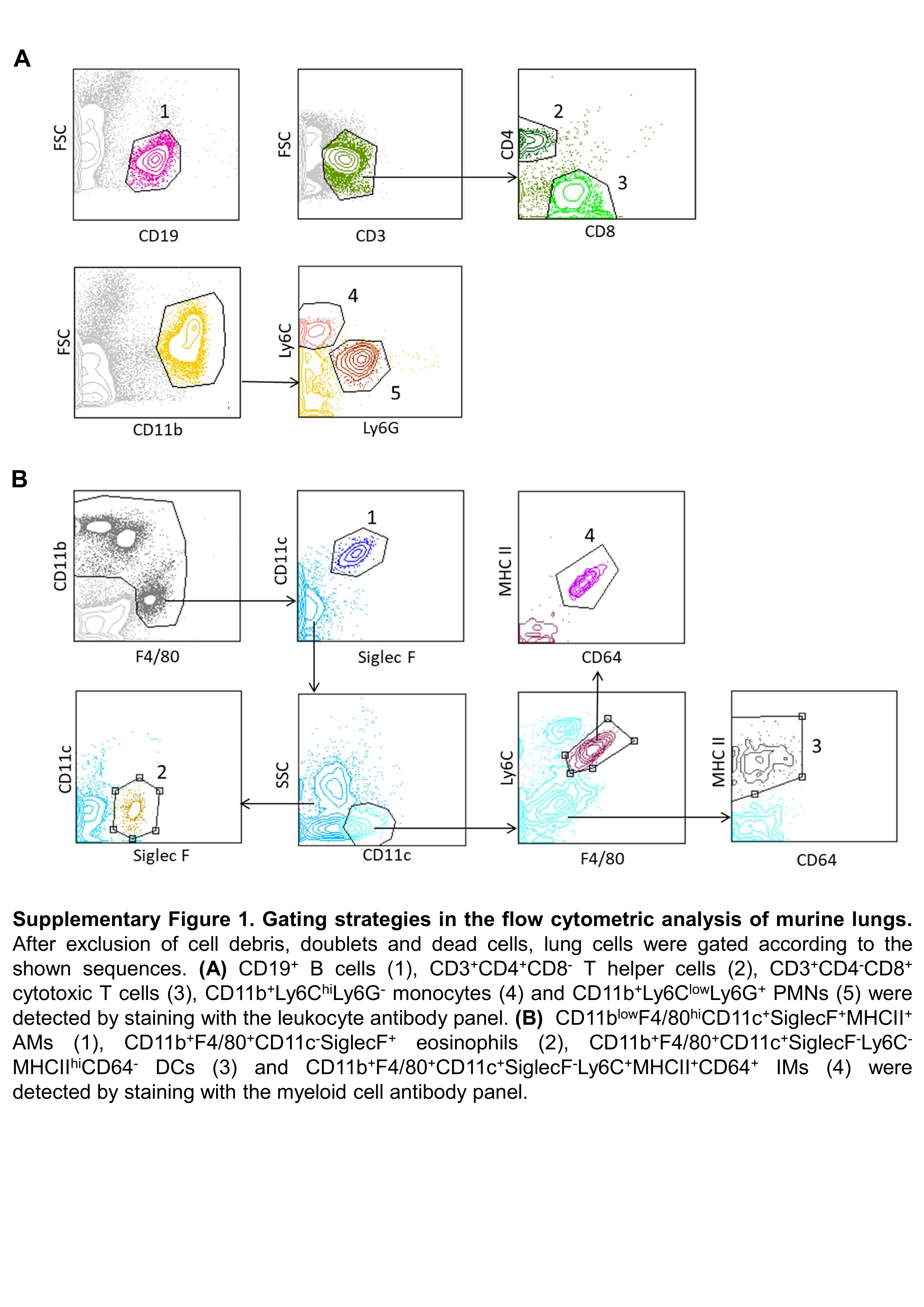

Supplement: Supplementary file 4 [file Image_1.jpeg]

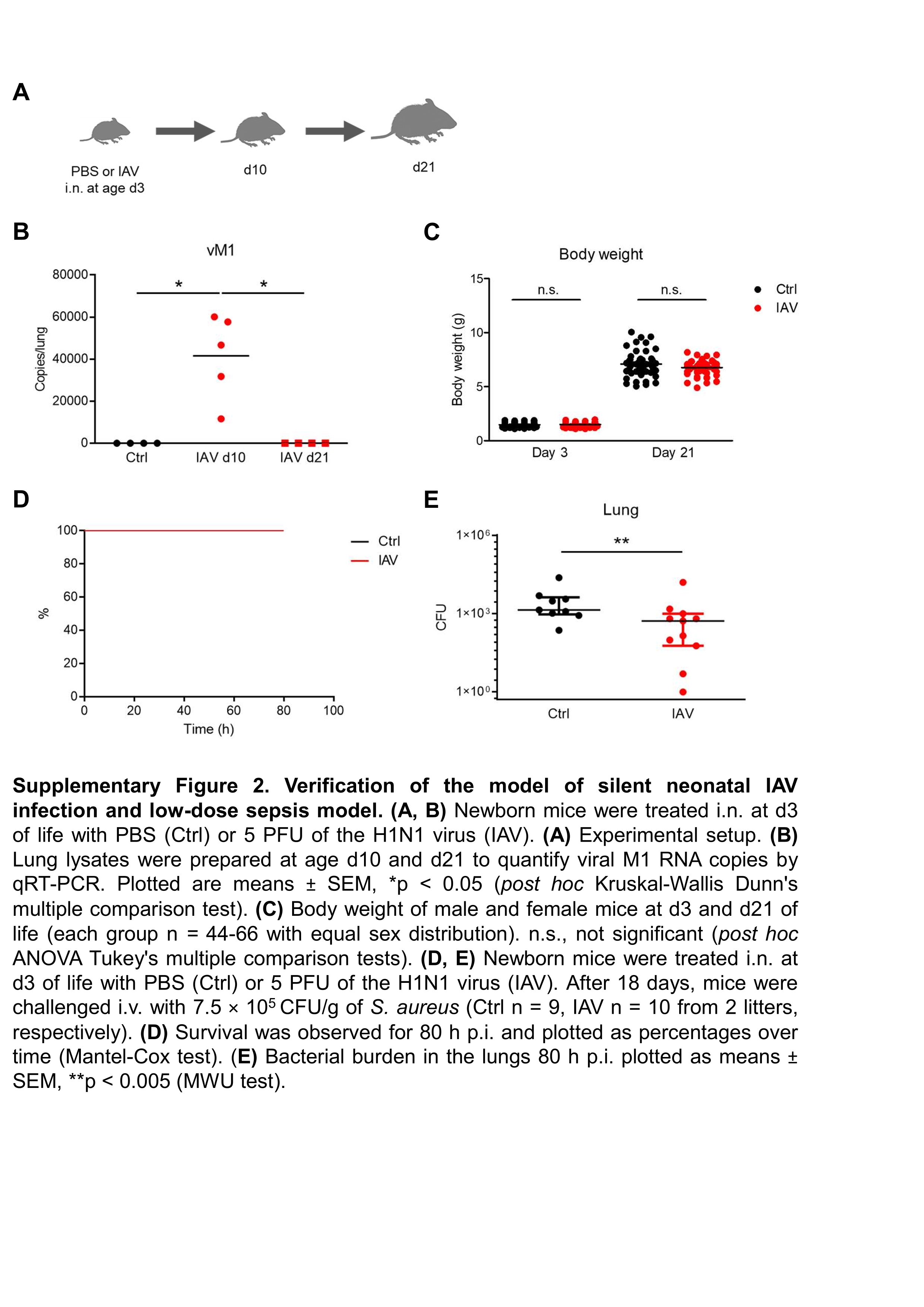

Supplement: Supplementary file 5 [file Image_2.jpeg]

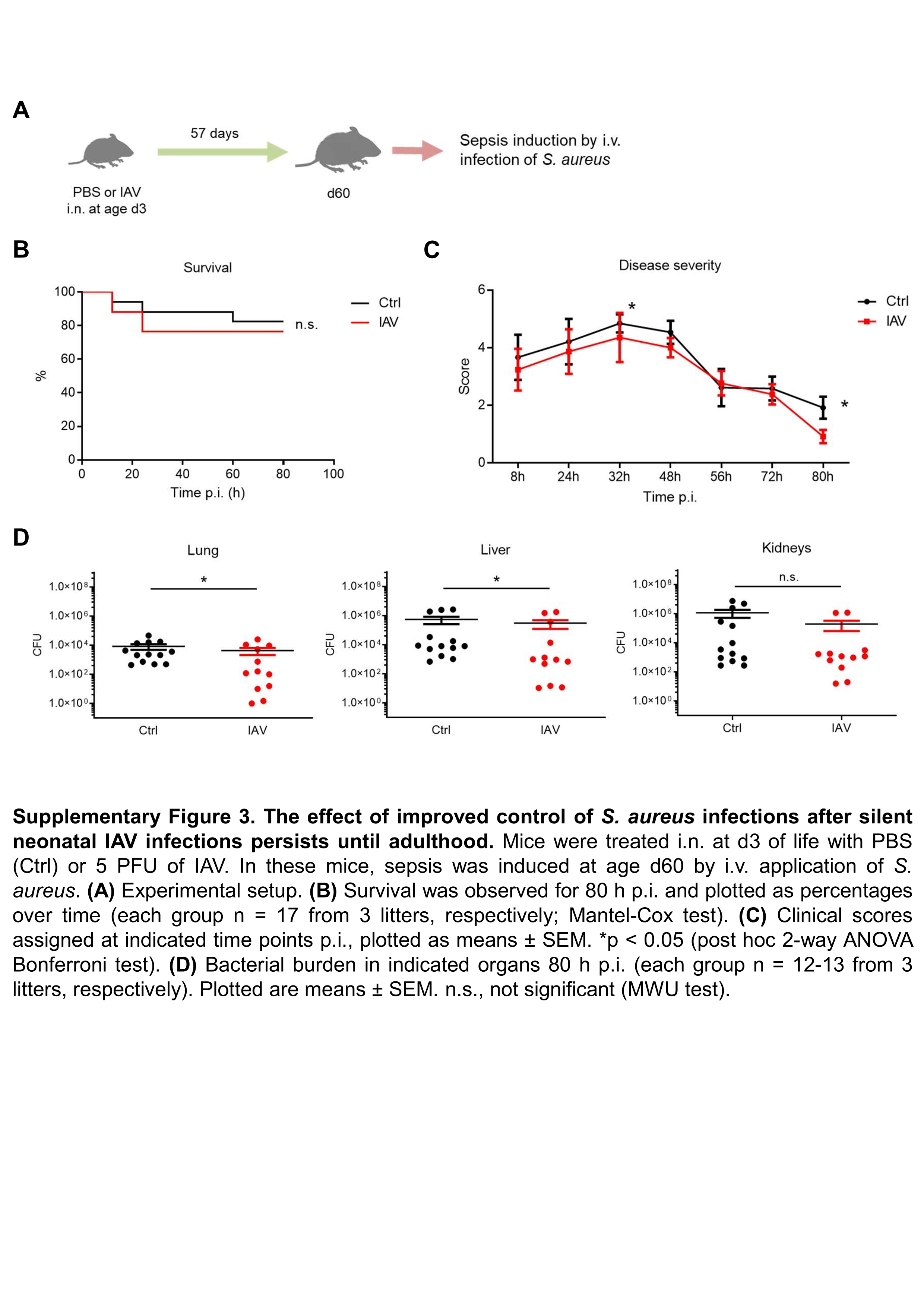

Supplement: Supplementary file 6 [file Image_3.jpeg]

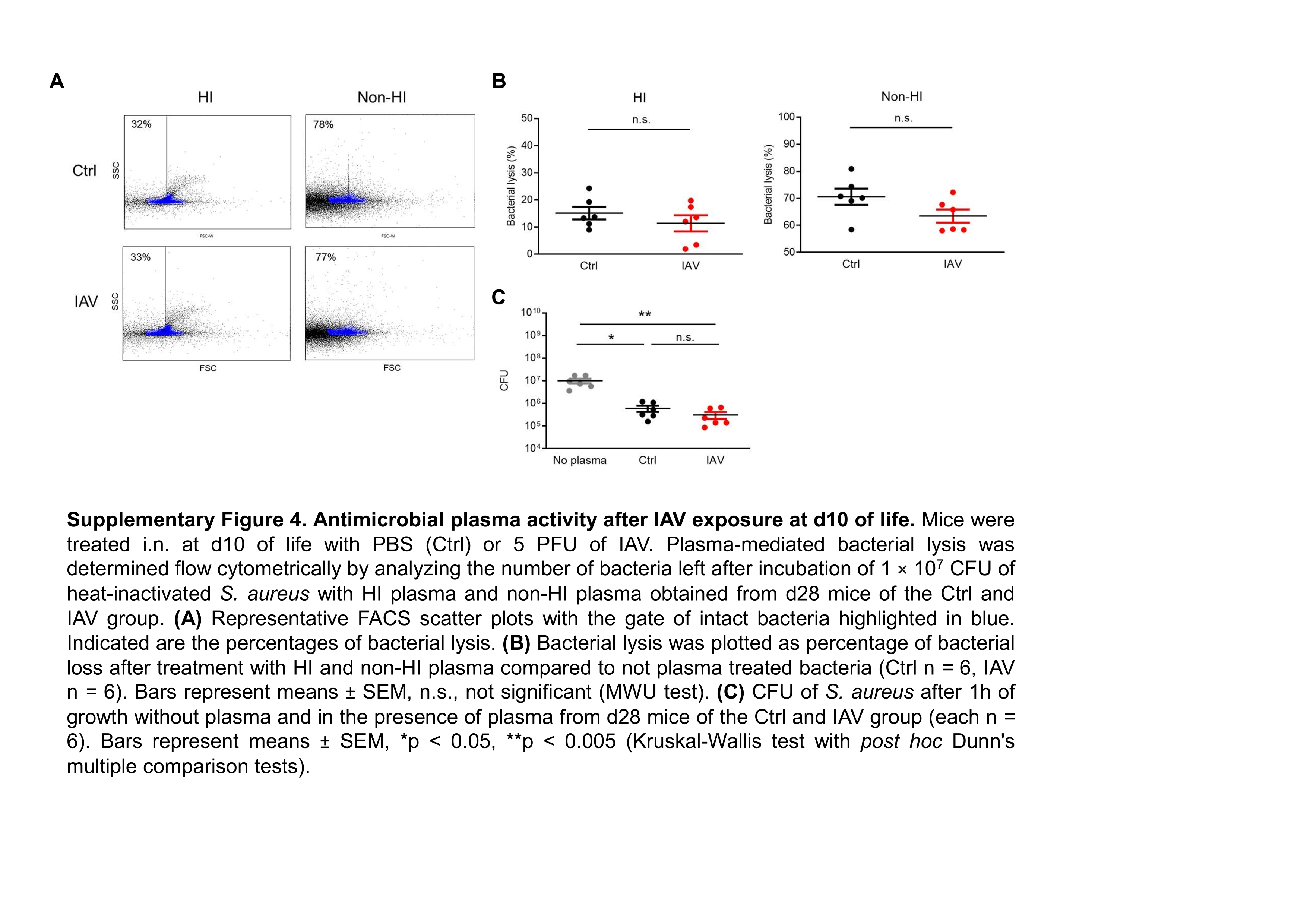

Supplement: Supplementary file 7 [file Image_4.jpeg]
